# Association between Polymorphism of Exportin-5 and Susceptibility to Lead Poisoning in a Chinese Population

**DOI:** 10.3390/ijerph14010036

**Published:** 2016-12-31

**Authors:** Hengdong Zhang, Ming Xu, Qiuni Zhao, Kai Sun, Wei Gong, Qiaoyun Zhang, Baoli Zhu, Yan An

**Affiliations:** 1 Department of Toxicology, School of Public Health, Jiangsu Key Laboratory of Preventive and Translational Medicine for Geriatric Diseases, Medical College of Soochow University, No. 199 Renai Road, Suzhou Industrial Park, Suzhou 215123, China; hd-zhang@263.net; 2 Institute of Occupational Disease Prevention, Jiangsu Provincial Center for Disease Control and Prevention, Nanjing 210028, China; sosolou@126.com (M.X.); fat3760@163.com (W.G.); qycdc@163.com (Q.Z.); zhbl5888@sina.com.cn (B.Z.); 3 School of Public Health, Southeast University, Nanjing 210009, China; firetadeas@foxmail.com; 4 Department of Emergency Medicine, the First Affiliated Hospital of Nanjing Medical University, Nanjing 210009, China; sunkai1212@126.com

**Keywords:** lead-sensitive and resistant, blood leads level, *XPO5* polymorphism, miRNA regulation

## Abstract

Lead (*Pb*) is one of the major contaminants in many industries, and imposes hazardous effects on multiple human organs and systems. Studies have shown that lead is able to induce the alteration of microRNA (miRNA) expression in serum and organs. In this study we investigated whether polymorphisms in miRNA-regulating genes were associated with the risk of lead exposure. We genotyped seven single-nucleotide polymorphisms (SNPs) in 113 lead-sensitive and 113 lead-resistant lead-related Chinese workers by Taqman analysis. The lead-sensitive group showed a significantly higher blood lead level (BLL) than the resistant group based on unconditional logistic regression results. One SNP in *XPO5* extron (rs2257082) was significantly associated with lead-poisoning (*p* = 0.022, odds rate (OR) = 1.63, 95% confidence interval (CI) = 1.07–2.47 in the C allele compared to the T allele). There were no significant associations between the other six SNPs and the blood lead levels. Therefore, polymorphism rs2257082 could be used to distinguish lead-resistant and lead-susceptible populations, and to develop more specific and accurate preventions.

## 1. Introduction

Lead (*Pb*) poses an enormous risk to human health due to its wide distribution in the environment and its extensive use in various industries. It has been demonstrated that acute and chronic exposure to lead has irreversible toxicity in several human organs and systems, such as the nervous, hematopoietic, reproductive systems, as well as in the kidney and bones [1,2]. Even a low blood lead level (BLL) (<10 μg/dL) was associated with kidney dysfunction in adults [3,4]. Developmental neurotoxicity has been observed for blood lead concentrations lower than 10 μg/dL in children [5,6]. Based on the known and established toxic effect, inorganic lead was defined as a potential human carcinogen (group 2A) by the International Agency for Research on Cancer (IARC). Therefore, there is an emergent need to identify valid biomarkers for predicting and preventing lead poisoning.

MicroRNAs (miRNAs), a family of small non-coding RNAs (~22 nucleotides), regulate post-transcriptional gene expression by binding to complementary sequences in the 3′-untranslated region (3′-UTR) of target messenger RNA (mRNA), and give rise to the silence of genes [7]. Up to 30% of protein-coding genes could be regulated by miRNAs, although miRNAs constitute only 1%–3% of the entire human genome [8]. Aberrant expression of miRNAs is related to various human diseases, such as hepatocellular carcinoma, lung cancer and endocrine pancreatic tumor [9]. Lead poisoning was also identified to be associated with dysregulated miRNA expression levels. In animal models, the altered expression levels of several miRNAs, which target crucial epigenetic mediators and neurotoxic proteins, were observed with early postnatal exposure to lead [10]. Lead-induced up-regulation of mir-203 could reduce the expression of the tricellulin protein and lead to blood–cerebrospinal fluid barrier loss as well [11]. Although studies have shown that the toxicity of lead is associated with miRNA alterations, the mechanisms of the variations of miRNAs were not comprehensively investigated and understood.

The biogenesis of miRNAs in mammalian cells involves both nuclear and cytoplasmic processing, beginning with the synthesis of primary miRNA (pri-mRNA) by RNA polymerase II [12]. The stem-loop structures contained in the primary transcripts are cleaved by the nuclear Drosha and its RNA-binding partner DGCR8, releasing a 60–70 nt hairpin, termed precursor-miRNA (pre-miRNA) [13]. The pre-miRNA generated in the nucleus is recognized and transported to the cytoplasm by the complex composition of exportin-5 (*XPO5*) and RAN-GTP [14]. Then, cytoplasmic Dicer processes pre-miRNA into a double-stranded RNA of ~22 nt and one strand of the duplex remains as mature miRNA [13]. Therefore, *XPO5* is a key element of the miRNA biogenesis pathway [15].

Single-nucleotide polymorphisms (SNPs) in miRNA genes, miRNA binding sites, and miRNA processing machinery, known as miR-SNPs, are able to alter the activity and expression of miRNA and the target genes, which eventually impact the development and prognosis of disease [16,17]. Aberrant expressions of *XPO5* occur in tumors, causing the accumulation of pre-miRNAs in the nucleus and damaging the production of mature miRNAs in cancer cells. To the best of our knowledge, the role of miR-SNPs regarding lead poisoning has not been well studied. Nevertheless, lead exposure accounts for the aberrant expression of miRNAs [18], and thus we hypothesize that miR-SNPs are strongly associated with lead poisoning. Our pioneer study explores whether polymorphisms in the miRNA machinery genes *GEMIN4*, *PIWIL1*, *RAN*, *DICER*, *DROSHA*, and *XPO5* are associated with lead toxicity in occupational workers exposed to lead.

## 2. Materials and Methods

### 2.1. Study Population

The study population consisted of 1130 workers under similar external lead exposure dose (0.017 ± 0.004 mg/m^3^) from five battery factories in Jiangsu Province, China. All workers started their lead-related works since 2012, each of whom had an orientation health check. All workers were initially healthy without aberrant BLL. Participants were excluded with evidence of any history of hematological disorders, liver or kidney dysfunction, or exposure to the medicine containing lead in daily life. Each participant was interviewed by a trained staff with standardized questionnaire, which included information about demographic characteristics, detailed occupational history, medical history, individual habits and self-conscious symptoms. In this study, we retrieved the physical examination data and survey data in the third years of each worker to make sure there was no different in their working age. We ranked participants’ severities of lead exposure based on their BLLs. Then we selected 10% individuals with the lowest BLLs as the most lead-resistant participants, while 10% with the highest BLLs as the most lead-sensitive ones. Each participant signed an informed consent. This research was approved by the Ethics Committee of the Jiangsu Province Center for Disease Control and Prevention (No. 2015025, 18 July 2012).

### 2.2. Blood Lead Levels Measurement

The 5 mL blood samples were collected in metal-free vacuum blood collection tube and stored at −4 °C for transportation. After the collection of blood samples, we finished the detection of BLLs in 48 h in order to reduce the interference for the BLL. Before the measurement, 0.2% nitrate acid was added into sample for further reaction which was necessary to our final measurement.

BLLs were measured by atomic absorption spectrometry using the PerkinElmer model 5000 graphite furnace atomic absorption spectrophotometer (PerkinElmer, Waltham, MA, USA). According to the Chinese standard, the standard substances of GBW09139h-09140h and GBW (e) 09054b-09056b were contained for each measurement of BLLs as controls. Each measurement was repeated by three persons independently in a blind fashion, and BLLs of samples with less than 5% concentration error were considered as qualified.

### 2.3. DNA Extraction

Approximately 5 mL venous blood sample was drawn from each participant into tubes containing EDTA and centrifuged immediately at 3000× *g* for 5 min to separate plasma and serum. DNA was extracted from the plasma by the QIAcube HT Plasticware and QIAamp 96 DNA QIAcube HT Kit (Qiagen, Dusseldorf, Germany) following the manufacturer’s protocol and then stored at −80 °C until use. The A260/A280 of the purified DNA, tested by Nanodrop OneC Ultramicro ultraviolet spectrophotometer (Thermo Scientific, Waltham, MA, USA), was between 1.8 and 2.0, indicating that there was no external contamination.

### 2.4. SNP Selection and Genotyping

miR-SNPs were selected based on the HapMap database, NCBI database and previous literature. The selection criterion was MAF (minor allele frequency) of HCB > 0.05 and in potential functional region of gene. The SNPs, which were reported in previous studies, were also included. *DICER* rs3742330 and rs13078, *DROSHA* rs6877842 and rs10719, *RAN* rs14035, *XPO5* rs2257082 and rs11077, *GEMIN4* rs910924, rs3744741, rs4968104 and rs2740348, *PIWIL1* rs1106042 were initially selected. After genotyping, *DICER* rs13078, *DROSHA* rs6877842, *XPO5* rs11077, and *PIWIL1* rs1106042 were excluded because the numbers of participants carrying the minor alleles were less than 10, which was unfeasible for reliable statistical analysis.

Genotyping of the selected SNPs was conducted by the ABI TaqMan SNP genotyping assays (Applied Biosystems, Foster City, CA, USA). The extracted DNA and genotyping assays were added to TaqMan universal PCR master mix (Applied Biosystems, Foster City, CA, USA) according to the manufacturer’s protocols. The genotyping procedures were further performed by ABI 7900 real-time PCR system (Applied Biosystems, Foster City, CA, USA). The condition for real-time PCR was as follows: 95 °C, 10 min; 95 °C, 15 s; 60 °C, 1 min (40 cycles of the last two steps). The data were analyzed via ABI 7900 System SDS 2.4.

### 2.5. Statistical Analysis

Statistical analysis was performed in SPSS 23.0 (Chicago, IL, USA). Hardy–Weinberg equilibrium was checked by goodness-of-fit χ^2^ test among resistant and the sensitive participants. Categorical variables were presented as percentages and continuous variables as mean ± SD (standard deviation). Student’s *t*-test was applied to differentiate the two groups for BLLs, while the differences in individual characteristics, such as age and gender, were compared by Pearson’s χ^2^. In this study, workers who smoked once a day for one year were defined as smokers, while individuals who had consumed alcohol once per week for one year were drinkers. To assess allele and genotype frequencies, we adjusted the degree of education (described in Table 1) and drinking status and further conducted a Student’s *t* test. Unconditional multivariate logistic regression was applied to compute odds ratio (OR) and 95% confidence interval (95% CI) for different genotypes. All probability measures corresponding to statistical significance were two-tailed (*α* = 0.05) and *p* < 0.05 was adopted as the criterion for statistical significance.

## 3. Results

### 3.1. Characteristics of Study Participants

The basic characteristics of the participants are presented in Table 1. No significant differences were detected between sensitive workers and resistant workers for gender (*p* = 0.286), BMI (*p* = 0.289), smoking status (*p* = 0.310) and drinking (*p* = 0.078). The participants’ educational level and eating or drinking habit (whether or not they do it in the workplace) were also similar (*p* = 0.412 and 0.847). However, lead-sensitive participants were marginally older than the lead-resistant ones (*p* = 0.047). BLLs of sensitive participants (the top 10% in BLL) and resistant participants (bottom 10% in BLL) were 513.86 ± 60.17 and 87.31 ± 20.54 μg/L, respectively.

### 3.2. Association between miR-SNPs and Plumbism

Table 2 demonstrates the genotypes and allele frequencies of miR-SNPs in sensitive and resistant participants adjusted by age, gender, education, and alcohol drinking. There is no deviation from the Hardy–Weinberg equilibrium (HWE) in the distributions of genotypes (*p* = 0.437). *XPO5* rs2257082 is the only SNP (out of the seven total SNPs) that is significantly different between sensitive and resistant participants: the T allele dominates in both sensitive and resistant workers (55.8% and 65.5%, respectively). Compared to the lead-resistant individuals, the C allele is more frequent in the lead-sensitive individuals, and is identified as a potential risk factor (44.2% vs. 34.5%, *p* = 0.022, OR = 1.63, 95% CI = 1.07–2.47). Furthermore, the CT/CC carriers have nearly twice the risk of internal lead exposure among sensitive individuals than the resistant ones (*p* = 0.038, OR = 1.85, 95% CI = 1.03–3.32). No other SNP shows a significant difference between the sensitive and resistant participants.

## 4. Discussion

In this study, we performed a genetic association analysis on the *XPO5* miR-SNP between participants with higher and lower occupational internal exposures to lead. We discovered that *XPO5* polymorphism is strongly associated with the susceptibility to lead poisoning, which backed our hypothesis that SNPs in miRNA-related genes could be associated with lead toxicity in occupational workers exposed to lead and implied that the C carriers of rs2257082 were more susceptible to occupational internal exposure to lead.

*XPO5* is a member of the karyopherin *β* family that takes advantage of RAN-GTP to control the nucleus export of per-miRNAs [14]. miRNAs are critical players in cellular processes, and are involved in cell development, proliferation, differentiation, and apoptosis by regulating the expression of target genes [12]. Therefore, the altered expression of miRNAs contributes to a wide spectrum of human diseases. miRNAs could also act as tumor suppressors or oncogenes [19]. miRNA expression patterns are found to be different between lead-exposed and non-exposed animals, associated with neurotoxicity [20], Alzheimer’s disease and blood–cerebrospinal fluid barrier loss [11]. It has been shown that down-regulated miRNA expression results from *XPO5* deletions [21]. In our previous study, we identified miR-520c-3p, miR-211 and miR-148a being aberrantly expressed in the lead-sensitive group involved in the present studies (unpublished data), and we hence hypothesized that the *XPO5* miR-SNP would be an influencing factor of occupational internal exposure to lead by altering miRNA expression. It is possible that the mutations of *XPO5* isolate the pre-miRNAs in the nucleus, restraining the expression of miRNAs, and further impede the miRNA biogenesis and function. The *XPO5* protein traverses the nuclear envelope [15], and is supposed to mediate the nuclear export of Dicer mRNA, one key component responsible for the cleavage of pre-miRNA to the mature miRNA [22]. We conjecture that *XPO5* miR-SNP may interact with Dicer, decline the expression of miRNAs, and thus interfere with miRNA biogenesis.

rs2257082 and rs11077 are the only two *XPO5* polymorphisms known for their significance in diseases. They are located on the extron and 3′UTR region of the *XPO5* gene, respectively. As a synonymous codon variant, rs2257082 is related to ovarian cancer [23] and idiopathic primary ovarian insufficiency [24]. Considering its special location and synonymous coding characteristics, the rs2257082 polymorphism most possibly affects mRNA structure, similar to other synonymous SNPs, which might influence mRNA folding [25] and stability [26], and eventually alter the translation rate of *XPO5*. In our study, we report that workers harboring the C allele tend to have higher BLLs. However, the specific molecular mechanism on how this SNP modifies the susceptibility to lead is not yet known; thus, further functional studies on rs2257082 will be necessary. The other focal SNP, rs11077, is related to non-small cell lung cancer (NSCLC) [27], colorectal cancer [28], multiple myeloma [29], Hodgkin’s lymphoma (HL) [30] and hepatocellular carcinoma (HCC) [31]. Unfortunately, as a low-frequency SNP in our study, participants carrying rs11077 AC/CC genotypes are rare (<10), and it might impact the accuracy of the statistical analysis. Future research with a considerably large sample size for lead-exposure participants is expected to study the accurate mechanism of rs11077 and its combined effect with rs2257082 on occupational internal exposure to lead.

## 5. Conclusions

In conclusion, our study is the first to investigate the relationship between miR-SNP and miRNA processing machinery in occupational subjects exposed to lead. We demonstrate that detection of the rs2257082 C allele was more frequent among more highly exposed workers.

## Figures and Tables

**Table 1:** The characteristics of the 10% most lead-sensitive and 10% most lead-resistant groups.

Characteristics	Group		*p*
	Lead-Resistant (*n* = 113) *n* (%)	Lead-Sensitive (*n* = 113) *n* (%)	
**Gender**			0.506
Male	52 (46.0)	57 (50.4)	
Female	61 (54.0)	56 (49.6)	
**Age (years)**	35.86 ± 10.26	38.39 ± 8.85	0.047 *
**BMI (kg/m^2^)**	23.7 ± 3.6	24.3 ± 4.8	0.289
**Smoking**			0.246
No	83 (73.4)	75 (66.4)	
Yes	30 (26.6)	38 (33.6)	
**Education**			0.412
Literate and up to lower secondary level	21 (18.6)	26 (23.0)	
Low up to middle secondary level	92 (81.4)	87 (77.0)	
**Drinking**			0.080
No	93 (82.3)	82 (72.6)	
Yes	20 (17.7)	31 (27.4)	
**Eat or drink in workplace**			0.847
No	31 (27.4)	30 (26.6)	
Occasionally	35 (31.0)	39 (34.5)	
Yes	47 (41.6)	44 (38.9)	
BLL (μg/L) *			<0.001 *
Mean ± SD	89.34 ± 15.39	513.52 ± 63.86	

BMI, body mass index; BLL, blood lead level. * *p*-value of two-sided Student’s *t*-test for age and BLL.

**Table 2:** Genotype frequencies of miR-SNPs in lead-sensitive and lead-resistant participants.

Genotype	Lead-Sensitive (*n* = 113)		Lead-Resistant (*n* = 113)		HWE	*p* *	Adjusted OR (95% CI) *
	*n*	%	*n*	%			
***DICER***							
**rs3742330**					0.430		
AA	47	41.6	51	45.1			1.00 (Ref.)
AG	46	40.7	47	41.6		0.957	0.98 (0.55–1.77)
GG	20	17.7	15	13.3		0.385	1.43 (0.64–3.18)
A allele	140	61.9	149	65.9			1.00 (Ref.)
G allele	86	38.1	77	34.1		0.488	1.14 (0.78–1.66)
***XPO5***							
**rs2257082**					0.543		
TT	32	28.3	47	41.6			1.00 (Ref.)
CT	62	54.9	54	47.8		0.096	1.68 (0.91–3.10)
CC	19	16.8	12	10.6		0.034	2.60 (1.08–6.28)
CT/CC	81	71.7	66	58.4		0.038	1.85 (1.03–3.32)
TT/CT	94	83.2	101	89.4			1.00 (Ref.)
CC	19	16.8	12	10.6		0.112	1.92 (0.86–4.30)
T allele	126	55.8	148	65.5			1.00 (Ref.)
C allele	100	44.2	78	34.5		0.022	1.63 (1.07–2.47)
***DROSHA***							
**rs10719**					0.104		
TT	90	79.6	83	73.4			1.00 (Ref.)
CT	23	20.4	30	26.6		0.563	0.83 (0.43–1.66)
CC	0	0.0	0	0.0		-	-
T allele	203	89.8	196	86.7			1.00 (Ref.)
C allele	23	10.2	30	13.3		0.563	0.83 (0.43–1.66)
***RAN***							
**rs14035**					0.080		
CC	65	57.5	81	71.7			1.00 (Ref.)
CT	48	42.5	32	38.3		0.061	1.73 (0.98–3.08)
TT	0	0.0	0	0.0		-	-
C allele	178	78.8	194	85.8			1.00 (Ref.)
T allele	48	21.2	32	14.2		0.061	1.73 (0.98–3.08)
***GEMIN4***							
**rs910924**					0.207		
CC	82	72.6	89	78.8			1.00 (Ref.)
CT	31	27.4	24	21.2		0.235	1.47 (0.78–2.78)
TT	0	0.0	0	0.0		-	-
C allele	195	86.3	202	89.4			1.00 (Ref.)
T allele	31	13.7	24	10.6		0.235	1.47 (0.78–2.78)
**rs4968104**					0.118		
TT	80	70.8	84	74.3			1.00 (Ref.)
AT	33	29.2	29	25.7		0.663	1.14 (0.63–2.08)
AA	0	0.0	0	0.0		-	-
T allele	193	85.4	197	87.2			1.00 (Ref.)
A allele	33	14.6	29	12.8		0.663	1.14 (0.63–2.08)
**rs2740348**					0.338		
CC	80	70.8	87	77.0			1.00 (Ref.)
CG	26	23.0	23	20.4		0.602	1.20 (0.61–2.33)
GG	7	6.2	3	2.6		0.238	2.36 (0.57–9.82)
C allele	186	82.3	197	87.2			1.00 (Ref.)
G allele	40	17.7	29	12.8		0.246	1.35 (0.81–2.23)

* Adjusted for sex, age, smoking, and education, drinking, and eating habit in workplace. *DICER*: dicer 1 ribonuclease III; *XPO5*: exportin 5; *DROSHA*: drosha ribonuclease III; *GEMIN4*: gem nuclear organelle associated protein 4.
